# BIDIRECTIONAL LINKS OF DAILY SLEEP QUALITY AND DURATION WITH PAIN AND SELF-RATED HEALTH IN OLD AGE

**DOI:** 10.1093/geroni/igac059.012

**Published:** 2022-12-20

**Authors:** Anna Jori Lücke, Cornelia Wrzus, Ute Kunzmann, Denis Gerstorf, Martin Katzorreck, Christiane Hoppmann, Oliver Schilling

**Affiliations:** Heidelberg University, Heidelberg, Baden-Wurttemberg, Germany; Ruprecht Karls University Heidelberg, Heidelberg, Baden-Wurttemberg, Germany; Leipzig University, Leipzig, Sachsen, Germany; Humboldt Universität zu Berlin, Berlin, Berlin, Germany; University of Leipzig, Leipzig, Sachsen, Germany; University of British Columbia, Vancouver, British Columbia, Canada; University of Heidelberg, Heidelberg, Baden-Wurttemberg, Germany

## Abstract

Sleep and physical well-being (e.g., pain, self-rated health) are closely linked, but the temporal ordering, especially regarding day-to-day variations, is not well understood. Furthermore, sleep quality and duration are only moderately correlated and may differ in their association with physical wellbeing. Using data from 123 young-old (66-69 years, 47% women) and 47 old-old adults (84-90 years, 60% women) who rated sleep quality and duration as well as pain and self-rated health on seven consecutive days, we examined bidirectional links between sleep and physical well-being. Supporting our hypotheses, results showed that after longer and better than usual sleep, participants reported better self-rated health; only better sleep quality significantly predicted lower pain. In turn, both lower pain intensity and increased self-rated health predicted better subsequent sleep quality, but not duration. We discuss conceptual and practical implications of our findings.

